# Upfront stereotactic radiosurgery for large posterior fossa metastases: a multicenter evaluation of clinical outcomes

**DOI:** 10.1007/s11060-026-05483-w

**Published:** 2026-03-02

**Authors:** Ariel Ben-Shoshan, Sami Heymann, José Asprilla, Paz Kelmer, Samuel Moscovici, Yair Hillman, Noam Weizman, Rotem Bohbot, Anton Wohl, Zvi R. Cohen, Yaacov R. Lawrence, Marc Wygoda, Yigal Shoshan, Tehila Kaisman-Elbaz, Tal Falick Michaeli

**Affiliations:** 1 Department of Radiation Oncology, Sharett Institute of Oncology, Hadassah Medical Center, Jerusalem, 91120 Israel; 2 Department of Neurosurgery, Neurosurgical Oncology and Radiosurgery Unit, Sheba Tel HaShomer Medical Center, Ramat-Gan, Israel; 3 Department of Neurosurgery, Hadassah Medical Center, Jerusalem, 91120 Israel; 4 Azrieli Faculty of Medicine, Bar-Ilan University, Safed, Israel; 5 Faculty of Medical and Health Sciences, Tel Aviv University, Tel Aviv-Yafo, Israel; 6 Benjamin Davidai Department of Radiation Oncology, Sheba Medical Center, Ramat-Gan, Israel; 7 Department of Developmental Biology and Cancer Research, Institute for Medical Research Israel-Canada, Hebrew University Medical School, Jerusalem, 91120 Israel

**Keywords:** Posterior fossa metastases, Stereotactic radiosurgery (SRS), Large brain metastases, Cerebellar metastases, Local control, Overall survival

## Abstract

**Purpose:**

Management of large posterior fossa metastases is challenging due to proximity to critical neuroanatomical structures and the potential need for urgent surgical decompression. While surgical resection is traditionally favored for large or symptomatic lesions, the role of upfront stereotactic radiosurgery (SRS) remains incompletely defined. This study aims to evaluate the clinical, radiological, and survival outcomes of upfront SRS for large cerebellar metastases to assess its viability as a primary treatment.

**Methods:**

This retrospective, two-institution study (2007–2024) included 61 adults with large cerebellar metastases (volume ≥ 5 cm³). Primary outcomes were overall survival (OS), local control (LC), and subsequent neurosurgical intervention. Secondary outcomes included tumor volume reduction.

**Results:**

Mean tumor volume was 11.9 cm³. LC was achieved in 93% of evaluable patients. Median OS was 12.6 months, with estimated survival rates of 68%, 54%, and 43% at 6, 12, and 24 months, respectively. Significant tumor mass reduction occurred following SRS (mean decrease 6.16 cm³, *p* < 0.001). Only 5% of patients required post-treatment neurosurgery. On multivariable analysis, a greater number of treatment fractions was associated with improved survival, whereas higher total radiation dose was associated with worse survival.

**Conclusions:**

Upfront SRS for large posterior fossa metastases provides excellent LC, significant volume reduction, and favorable survival, while rarely necessitating subsequent surgical intervention. These findings suggest that, in carefully selected patients, SRS may serve as an effective primary treatment strategy that can obviate the need for surgical resection, even in large-volume posterior fossa disease.

**Supplementary Information:**

The online version contains supplementary material available at 10.1007/s11060-026-05483-w.

## Introduction

Brain metastases are a common and clinically significant complication of systemic cancer, occurring in up to 40% of patients with solid tumors [1–4]. Their management represents a complex oncologic challenge and typically requires a multidisciplinary approach. While surgical resection, whole-brain radiotherapy (WBRT), and stereotactic radiosurgery (SRS) are well-established treatment modalities for brain metastases, the optimal management strategy for large cerebellar metastases remains a subject of ongoing debate [4].

Large cerebellar metastases pose unique clinical challenges due to their proximity to critical structures such as the brainstem and the fourth ventricle [2]. Beyond absolute tumor size, anatomical orientation and the degree of fourth ventricle displacement are clinically relevant factors, as they may influence symptom development, the risk of hydrocephalus, and treatment decision making. Lesions in this location frequently produce mass effect, resulting in headaches, nausea and vomiting, gait instability, and ataxia, and may lead to significant neurological deterioration. Effective management requires integrated assessment of tumor size, anatomy, peritumoral edema, fourth ventricle compression, and clinical status [2].

Surgical resection has traditionally been considered the preferred treatment for large or symptomatic cerebellar metastases, aiming to achieve maximal safe resection and rapid relief of mass effect. However, surgery in the posterior fossa carries substantial risks, including leptomeningeal dissemination, infection, hemorrhage, neurological deficits, and cerebrospinal fluid leakage, largely related to the complex regional anatomy [5]. WBRT is currently reserved mainly for patients in whom SRS is not feasible, such as those with limited life expectancy or extensive intracranial disease [6].

SRS offers a non-invasive alternative for selected cerebellar metastases, delivering high radiation doses with relative sparing of surrounding normal tissue. This approach is particularly attractive for patients who are poor surgical candidates or who harbor multiple brain metastases [7–8]. Previous studies have reported favorable local control and survival outcomes following SRS, including in large cerebellar lesions [7–8]. A meta-analysis demonstrated a one-year local control rate of approximately 85% for metastases measuring 4–14 cm³ treated with SRS [9]. Chang et al. reported a median overall survival of 12 months, with one- and two-year survival rates of 52% and 25%, respectively, in patients with large cerebellar metastases treated with SRS [10]. Although SRS is generally well tolerated, treatment-related complications such as radiation induced edema and necrosis may occur and are usually managed medically, while severe complications requiring surgical intervention have been reported in approximately 5–15% of cases [11–12].

Despite these advances, surgery remains a cornerstone in the management of large, symptomatic cerebellar metastases. Surgical series have reported one-year local control rates of 70–80% and a median overall survival of approximately 15 months [13]. However, these outcomes are accompanied by relatively high rates of severe complications, ranging from 10% to 30%, exceeding those typically observed after SRS [13–15].

In the context of evolving multidisciplinary strategies for the treatment of large brain metastases, integrating surgery, radiotherapy, and systemic therapies, the role of upfront stereotactic radiosurgery for large volume cerebellar metastases remains to be clearly defined [16]. While data on SRS for brain metastases are abundant, evidence specifically regarding large-volume lesions in the posterior fossa remains limited. This two-institution retrospective study evaluates upfront SRS for posterior fossa metastases with a tumor volume greater than 5 cm³, as a primary strategy to safely spare patients from the significant morbidity and surgical risks associated with posterior fossa resection, even in the setting of large-volume disease.

## Methods

### Study design and patient population

This was a retrospective, two-institution cohort study including patients treated at Hadassah Ein Kerem Medical Center and Sheba Medical Center between 2007 and 2024. Eligible patients were aged 18 years or older, diagnosed with large cerebellar metastases originating from various primary malignancies, defined as a tumor volume of ≥ 5 cm³ on contrast-enhanced T1-weighted brain MRI, and treated with upfront SRS.

During the study period, many patients with large posterior fossa metastases underwent upfront surgical resection, particularly in cases requiring urgent decompression due to acute hydrocephalus or rapid neurological decline, and were therefore excluded from this cohort. The study population represents a selected group treated with upfront SRS, including patients considered poor surgical candidates due to systemic comorbidities or contraindications to general anesthesia, as well as those who declined surgery for personal reasons.

### Data collection

Clinical data were extracted from electronic medical records and included age, sex, primary tumor type, systemic disease status, number and location of brain metastases, performance status according to the Eastern Cooperative Oncology Group (ECOG) scale, treatment characteristics, post-treatment complications, and subsequent surgical interventions. Radiological data included tumor volume, peritumoral edema, and mass effect on the fourth ventricle, assessed on high-resolution contrast-enhanced T1-weighted brain MRI scans obtained before and after treatment. Tumor volumes were measured on pre- and post-SRS/FSRS MRI scans using Brainlab Elements Cranial SmartBrush software. Use of adjunctive therapies - including chemotherapy, immunotherapy, targeted or hormonal therapy, and WBRT, as well as post-treatment corticosteroid use was also recorded.

### Stereotactic radiosurgery treatment

All treatments were delivered using a linear accelerator (LINAC)-based stereotactic radiosurgery system (2005–2015: Varian DBX with Brainlab M3; 2016–2020: TrueBeam Novalis STx with ExacTrac X-Ray). Patients were treated with either single-fraction SRS or fractionated stereotactic radiosurgery (FSRS), according to multidisciplinary discussion and institutional consensus guidelines. Prior to 2016, rigid stereotactic frame fixation was used for single-fraction SRS, while a rigid thermoplastic, patient-specific mask was used for fractionated treatments. From 2016 onward, a rigid thermoplastic, patient-specific mask was used for both SRS and FSRS treatments [17].

Treatment planning was based on high-resolution computed tomography (CT) imaging fused with axial three-dimensional T1-weighted gadolinium-enhanced MRI (slice thickness 0.5–0.625 mm). In all patients, the MRI used for target delineation was acquired on the same day as the planning CT scan. The gross tumor volume (GTV) was defined as the contrast-enhancing lesion, without the addition of a planning target volume (PTV) margin. Radiation planning was performed using dynamic conformal arc and/or intensity-modulated radiotherapy (IMRT) techniques including Volumetric Modulated Arc Therapy (VMAT). The choice of fractionation scheme was determined primarily by tumor volume and proximity to critical OARs. Hypofractionated SRS was preferentially used for larger lesions or those adjacent to critical structures. No PTV margin was applied. Dosimetric parameters, including V12Gy/V18Gy, as well as brainstem constraints were evaluated before plan approval to ensure safe dose delivery to surrounding normal tissue. Special attention was given to organs at risk (OARs) in the posterior fossa, particularly the brainstem and fourth ventricle. Dose prescription and fractionation were individualized based on lesion size and proximity to critical structures, in accordance with dose constraints outlined in AAPM Task Group 101 (TG-101) [18]. For instance, in 3-fraction FSRS plans, the brainstem D_0.5cc_ was limited to ≤ 18 GY and D_max_ to ≤ 23.1 GY, as illustrated in our representative plans (Online Resource 1). Treatment planning aimed for optimal target coverage (typically > 95%) and conformality, with doses prescribed to the 80% isodose line. Dosimetric metrics, including the volume of brain receiving 12 Gy (V12Gy) for SRS and V18Gy for 3-fraction FSRS, were monitored during planning to ensure adherence to TG-101 brainstem constraints. Post-treatment follow-up consisted of contrast-enhanced brain MRI performed every 3 months according to institutional protocol.

### Outcome measures

The primary outcome measures were overall survival (OS), defined as the time from SRS to death from any cause; local control (LC), defined as the absence of progression at the treated site; and treatment-related complications requiring neurosurgical intervention (specifically resection of the treated metastasis or ventriculoperitoneal shunt placement). Secondary outcomes included radiological tumor mass reduction and ECOG performance status at follow up. Radiographic response and local control were assessed using criteria consistent with RANO-BM guidelines. Local failure was defined as an increase in tumor volume on follow-up MRI, while time-to-local failure (TTLF) was calculated from treatment completion. Dosimetric quality was evaluated using the Conformity Index (CI) and target coverage, with a median prescription to the 80% isodose line. For toxicity, the V12Gy (volume of brain receiving 12 Gy) was monitored as a metric for potential radionecrosis.

### Ethics approval

The study was approved by the institutional review boards (IRB) of both participating centers.

### Statistical analysis

Continuous variables are presented as mean ± standard deviation (SD), median, and range, while categorical variables are presented as frequencies and percentages. Associations between categorical variables were assessed using Fisher’s exact test. Comparisons of continuous variables between two independent groups were performed using the nonparametric Mann Whitney U test. Changes in continuous variables over time were assessed using paired t-tests. The effects of categorical variables on overall survival were evaluated using the Kaplan Meier method, with survival curves compared using the log-rank test. Cox proportional hazards regression models were used for multivariable survival analysis. Local control was assessed both by standard Kaplan Meier estimates and using the Cumulative Incidence Function (CIF) with the Fine-Gray model to account for death as a competing risk. All statistical tests were two-sided, and a p-value of ≤ 0.05 was considered statistically significant.

## Results

A total of 61 patients met the inclusion criteria and were included in the analysis. Mean age was 67.4 years (range, 41–90), with an approximately equal sex distribution (50.8% male and 49.2% female). Mean tumor volume on baseline MRI was 11.91 cm³ (range, 5–30). Lesions were located in the cerebellar vermis in 13 patients (21.3%) and in the cerebellar hemispheres in 48 patients (78.7%) (Table 1).

**Table 1:** Baseline characteristics of the study population

Characteristic	Value
**AGE**	67.37 ± 10.19
**SEX**	
Female	30 (49.2%)
Male	31 (50.8%)
**Performance status after 6 months (ECOG)** ^**a**^ **[** ***n*** ** = 20]**	
ECOG 0	9 (45%)
ECOG 1	6 (30%)
ECOG 2	2 (10%)
ECOG 3	3 (15%)
**Tumor Origin**	
Lung Cancer	33 (54.1%)
Breast Cancer	14 (23%)
GI tract Cancer	5 (8.2%)
Gynecological Cancer	3 (4.9%)
Other	6 (9.8%)
**Systemic Spread (PET-CT)**	
None, Only Brain	13 (21.3%)
Limited spread, 1–2 sites	37 (60.7%)
Multiple sites (3 or more)	11 (18%)
**Additional brain metastases**	
No	14 (23%)
Cerebrum	6 (9.8%)
Cerebellum	9 (14.8%)
Both	32 (52.5%)
**Treatments** ^**b**^ **[** ***n*** ** = 59]**	
Chemo	17 (28.8%)
Immuno	10 (16.9%)
Hormonal/Targeted	13 (22%)
Combo	15 (25.4%)
WBRT	1 (1.7%)
NONE	3 (5.1%)
**Metastatic volume Pre treatment (MRI**,** ml)**^**c**^**[*n* = 60]**	11.91 ± 6.66
**Metastatic location (MRI)**	
Vermis	13 (21.3%)
Cerebellar hemispheres	48 (78.7%)
**Mass effect on the 4th ventricle**	
Normal	28 (45.9%)
Mild	11 (18%)
Moderate	18 (29.5%)
Severe	4 (6.6%)
**Surgery after treatment** ^**c**^ **[** ***n*** ** = 60]**	
No	57 (95%)
Resection of metastasis	2 (3.3%)
VP Shunt	1 (1.7%)

Data are presented as Mean ± SD for continuous variables, and as number (percentage) for categorical variables

^a^ ECOG performance status was available for 20 patients. ^b^ Treatment data were available for 59 patients. ^c^ Data on pretreatment metastasis volume and the need for surgery after treatment were available for 60 patients

Primary origins included lung (54.1%), breast (23%), gastrointestinal (8.2%), and gynecological (4.9%). Systemic metastatic burden was diverse: 18% had widespread extracranial metastases, 60.7% had limited spread, and 21.3% had no systemic disease (Table 1). Regarding intracranial disease, 23% of patients had isolated cerebellar metastases, while 77% presented with additional intracranial disease elsewhere in the brain. 9.8% had additional cerebrum metastases, 14.8% had multiple cerebellar metastases, and 52.5% had both additional cerebellar and cerebral metastatic lesions. Radiologically, a moderate to severe mass effect on the fourth ventricle was observed in 36.1% of patients, with hydrocephalus present in 11.5% and cerebellar edema in 88.5% (Table 1).

Systemic treatments included chemotherapy (28.8%), immunotherapy (16.9%), and targeted or hormonal therapy (22%), while 25.4% received a combination of treatments. One patient (1.7%) received WBRT, and three patients (5.1%) did not receive any systemic treatment (Table 1). Systemic treatment information was not available for two patients.

Following SRS, the vast majority of patients (95%) did not require any further surgical intervention. Regarding neurosurgical interventions (5% total): only two patients (3.3%) underwent surgical resection due to symptomatic local progression at 10 and 12 months, and one patient (1.7%) required the placement of a delayed ventriculoperitoneal (VP) shunt for persistent symptomatic hydrocephalus and edema (Table 1). No cases of symptomatic radionecrosis were documented. Corticosteroids were administered in 93.4% of patients at the time of SRS, either for symptomatic management or prophylactically. Detailed information regarding the duration of steroid therapy or long-term use was not available. Baseline hydrocephalus (11.5%) and peritumoral edema (88.5%) were primarily managed with corticosteroids and upfront SRS, leading to symptomatic relief in the majority of patients. Functional status at six months was available for 32.8% of the cohort, with 75% demonstrating an ECOG score of 0–1 (Table 1).

Treatment characteristics for the 61 patients are detailed in Table 2. Twenty patients received single fraction SRS with a median marginal dose of 18 Gy (range, 18–25 Gy). Forty-one patients were treated with FSRS in 3 to 6 fractions (median, 4), with a median total dose of 28 Gy (range, 25–41.25 Gy). Dosimetric quality was maintained across the cohort through standardized planning protocols. In representative treatment plans (Online Resource 1), the V12Gy was 10.7 cm³ for a single-fraction SRS (20 Gy) and the target coverage was ≥ 99.8%. The mean Conformity Index (CI) for these representative cases was 1.19, reflecting the high precision of the LINAC-based delivery.

**Table 2:** Summary of treatment characteristics

Parameter	Single-fraction SRS (*n* = 20)	Fractionated SRS (FSRS) (*n* = 41)
Number of Fractions	1	3–6 (median 4)
Median Total Dose (Gy)	18	28
Total Dose range (Gy)	18–25	25-41.25
Proportion of patients	32.80%	67.20%

**Fig. 1:**
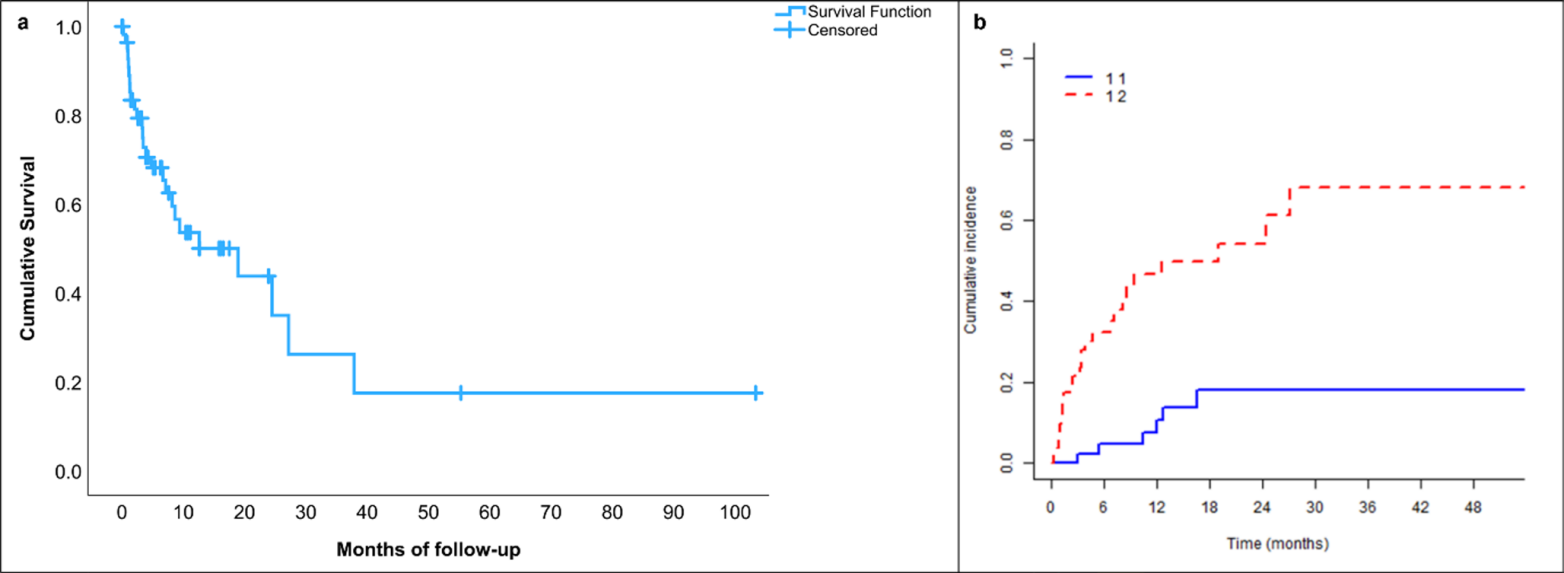
Survival and intracranial control outcomes following SRS. (**a**) The plot shows the cumulative overall survival of the study cohort over time, estimated using the Kaplan Meier method. The median OS was 12.6 months. Survival probabilities at key time points were approximately 68% at 6 months, 54% at 12 months, and 43% at 24 months. Censored observations are marked with a plus symbol (+). (**b**) Cumulative incidence of local failure with death as a competing risk. The solid blue line (11) represents the cumulative incidence of local failure, which remained low at approximately 5% at 6 months and 11% at 12 months. The dashed red line (12) indicates the cumulative incidence of death without local failure (competing risk), estimated at approximately 33% at 6 months and 50% at 12 months

Local control of the treated cerebellar lesions was achieved in 93% of evaluable patients, while 6.7% experienced radiological progression. Data on post-treatment MRI response were not available for 16 patients. In addition to the crude local control rate of 93%, an actuarial analysis using the Cumulative Incidence Function (CIF) was performed to account for death as a competing risk (Fig. 1). The cumulative incidence of local failure was 5% at 6 months and 11% at 12 months. The median OS for the cohort was 12.6 months (0.2-103.36). Estimated OS rates at specific time points were approximately 68% at 6 months, 54% at 12 months, and 43% at 24 months (Fig. 1).

To identify factors influencing survival, Kaplan Meier curves were stratified by systemic treatment modality. Patients receiving chemotherapy had a median OS of 6.67 months (95% CI: 0-14.58); those receiving immunotherapy, 7.13 months; and those treated with combination therapy, 27.17 months (95% CI: 0-54.35). However, these differences were not statistically significant (log-rank test, *p* = 0.286).

In the univariate Cox regression analysis, none of the evaluated baseline factors, including age, lesion volume, total radiation dose (Gy), and presence of systemic spread, were significantly associated with OS.

In the multivariate Cox regression model, treatment-related parameters emerged as the primary determinants of OS. Higher total radiation dose was independently associated with an increased risk of mortality (HR = 1.17, 95% CI: 1.01–1.35, *p* = 0.043), indicating that patients who received larger cumulative doses experienced poorer outcomes after adjustment for relevant covariates. In contrast, the number of fractions demonstrated a protective effect. Treatment delivered over a greater number of fractions was significantly associated with improved survival (HR = 0.53, 95% CI: 0.29–0.97, *p* = 0.038). A representative example of single fraction versus hypofractionated treatment planning is shown in Online Resource 1. Other variables, including age, lesion volume, systemic spread, presence of multiple intracranial lesions, and treatment type, were not significantly associated with survival in the multivariate model. An era analysis (2007–2015 vs. 2016–2024) revealed no statistically significant difference in OS, indicating consistent outcomes despite technological advancements.

**Fig. 2:**
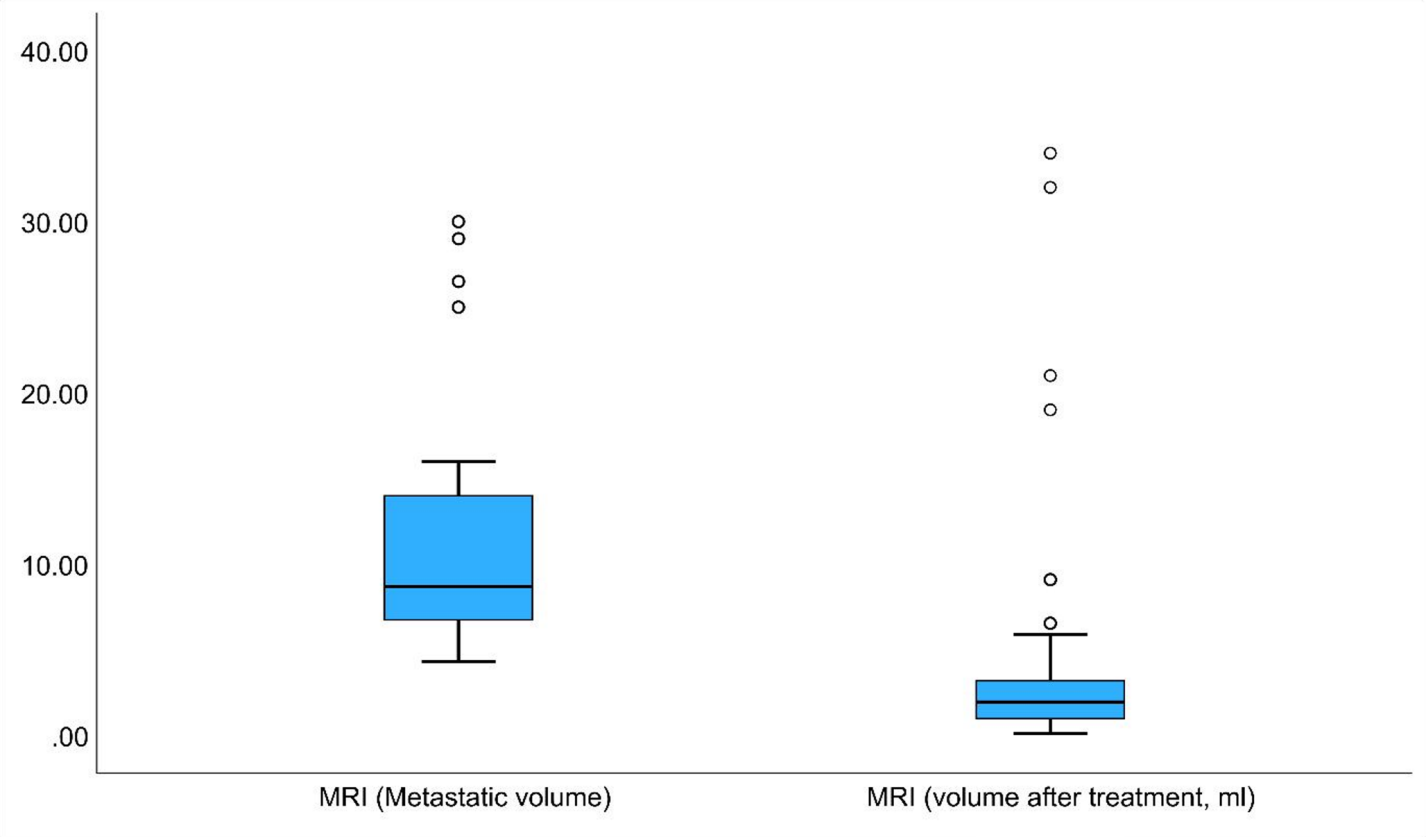
Boxplots of metastatic volume before and after SRS. Tumor volumes were measured on MRI before and after treatment, with post-treatment imaging performed within 3 months. A clear reduction in metastatic volume was observed following treatment. The mean pretreatment volume was 11.17 cm³ (SD = 6.89), and the mean post-treatment volume was 5.0 cm³ (SD = 8.50). Paired t-test analysis revealed a statistically significant reduction in volume (t (33) = 8.005, *p* < 0.001), with a mean difference of 6.16 cm³ (95% CI: 4.59–7.72)

**Fig. 3:**
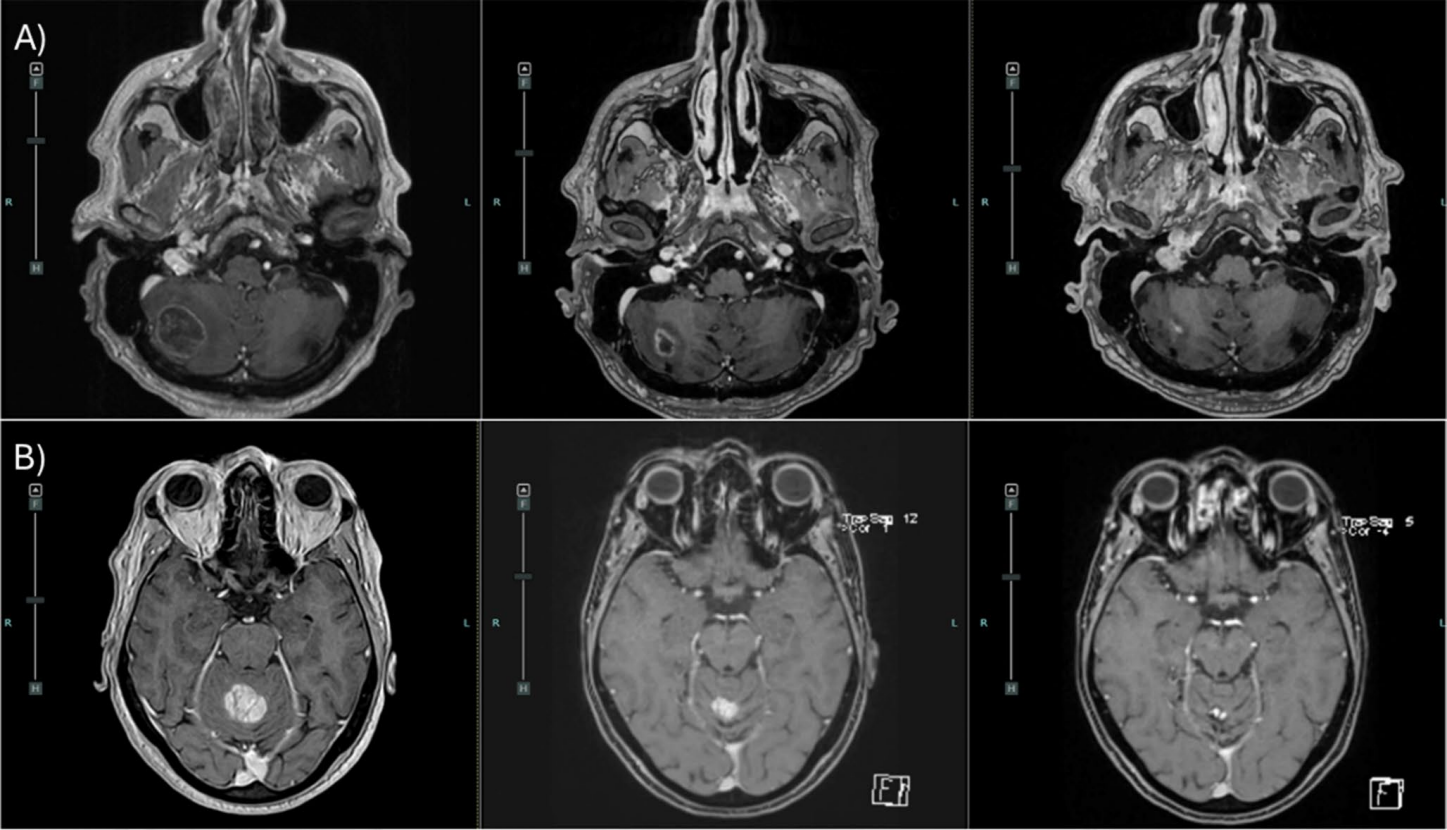
Axial post-contrast T1-weighted MRI scans showing cerebellar metastases and SRS treatment response. (**A**) A 75-year-old patient with a right cerebellar lesion (9.31 cm³) treated with single fraction SRS (20 Gy to the 80% isodose line). (**B**) A 58-year-old patient with a superior vermian lesion (6.3 cm³) treated with FSRS (28 Gy in 4 daily fractions to the 80% isodose line). Images are shown at baseline (left), 3 months (middle), and 6 months (right) post treatment

Finally, we evaluated the change in tumor volume after SRS in a subgroup of patients with available imaging. Pre-treatment volumetric MRI data were retrievable for 60 patients due to missing historical contouring data for one individual. The average tumor volume before treatment was 11.17 cc (SD = 6.89), which decreased to 5.0 cc (SD = 8.50) after treatment. This reduction was statistically significant according to a paired t-test (t (33) = 8.005, *p* < 0.001), with an average decrease in volume of 6.16 cc (95% CI: 4.59–7.72). These quantitative findings are illustrated in Fig. 2, which shows boxplots of metastatic volume before and after treatment, and in Fig. 3, which provides representative axial MRI scans demonstrating tumor response at baseline, 3 months, and 6 months post treatment.

## Discussion

This two-institution retrospective study demonstrates that SRS for large cerebellar metastases achieves excellent local control (93%), significant tumor mass reduction, and a median OS exceeding 12 months, with minimal neurosurgical intervention (5%). These findings strengthen the growing body of evidence supporting upfront SRS as an effective and safe treatment option for large posterior fossa lesions.

### Local control and survival outcomes

To more accurately evaluate intracranial efficacy, we performed a Cumulative Incidence Function (CIF) analysis, accounting for death as a competing risk. This showed a 12-month local failure rate of 11%, corresponding to an 89% actuarial local control rate, confirming the durable effect of upfront SRS. Despite a substantial competing risk of mortality (50% at 12 months), the stability of the local failure curve indicates that local disease in the posterior fossa is effectively controlled.

The median OS observed in our cohort (12.6 months) is consistent with previously published data on SRS for large cerebellar metastases. A comparable series reported a median OS of 10.5 months in patients with large cerebellar metastases from lung cancer, with 83.7% showing neurological improvement after SRS [19]. The close alignment of survival and functional outcomes across studies supports the robustness of SRS as a non-invasive therapeutic option for large lesions, including those located in eloquent or surgically challenging regions of the posterior fossa.

Similarly, previous reports demonstrated a median OS of 8.4 months and 98% local control following Gamma Knife radiosurgery (GKRS) for large posterior fossa metastases (≥ 2 cm) [20], and a median survival of 16.2 months and 90% local control using hypofractionated SRS (8 Gy × 3 fractions), with minimal severe toxicity [21]. The slightly longer survival observed in the latter study may reflect differences in fractionation schemes and patient selection, as their cohort excluded patients with poor systemic disease status. Taken together with our results, these data suggest that SRS provides durable intracranial control and comparable survival outcomes across heterogeneous patient populations.

Compared with surgical outcomes, SRS appears to offer similar oncologic efficacy with a more favorable safety profile. Previous surgical series reported one-year local control rates of 70–80% and median OS around 15 months following resection of cerebellar metastases, but with postoperative complication rates ranging from 10% to 30% [13–15]. In contrast, the low rate of severe adverse events (5–15%) in the SRS series, including ours (5%), underscores a potential safety advantage while maintaining equivalent disease control [20–21].

### Management of edema and mass effect

SRS effectively controlled peritumoral edema and relieved mass effect, achieving significant tumor reduction (mean decrease 6.16 cm³, *p* < 0.001) and improvement in mass effect with minimal need for subsequent surgical decompression (5%). Although baseline edema was high (88.5%), most patients improved with corticosteroids alone.

The significant volume reduction provides a ‘chemical decompression’ that alleviates mass effect on the fourth ventricle and brainstem. This directly mitigates the risks of acute hydrocephalus and neurological deterioration, as evidenced by our low rates of subsequent neurosurgery (3.3%) and VP shunt placement (1.7%).

These findings are consistent with previously published data, which reported median tumor shrinkage of approximately 59%, peritumoral edema reduction of nearly 80%, and relief of fourth ventricle compression following GKRS, with most patients showing symptomatic improvement and no requirement for urgent surgery [20, 22]. Similar outcomes were also observed in other cohorts, where over 80% of patients demonstrated neurological improvement following SRS for large cerebellar lesions [19].

Taken together, these studies, including ours, support the capacity of SRS not only to achieve tumor control but also to mitigate mass effect, improve neurological function, and reduce the need for emergent surgical intervention.

### Toxicity and safety

In our cohort, severe treatment- related complications were uncommon, with only three patients (5%) requiring neurosurgical intervention - two for tumor resection and one for delayed ventriculoperitoneal (VP) shunt placement. This distinction regarding the VP shunt is clinically significant, as its delayed nature indicates it did not reflect acute post-treatment obstructive hydrocephalus, but rather a later clinical development. This low complication rate is consistent with prior reports describing severe toxicity rates ranging between 5% and 15% following SRS for large cerebellar metastases [19–21]. While mild to moderate adverse effects, such as transient edema, steroid dependence, or headaches, were relatively frequent, they were generally manageable with medical therapy alone.

In contrast, surgical series have reported substantially higher complication rates, reaching up to 30%, including severe events such as hemorrhage, infection, leptomeningeal dissemination, and cerebrospinal fluid (CSF) leakage [13–15]. Furthermore, it is important to consider that according to current practice guidelines, surgically treated patients typically require adjuvant SRS to the resection cavity to optimize local control. Collectively, these considerations further support the role of SRS as a safe and effective alternative to upfront surgical resection, particularly for patients with limited systemic reserve, multiple brain metastases, or lesions located in surgically challenging regions of the posterior fossa.

### Prognostic factors

Our multivariable analysis highlights the critical role of treatment-related parameters in shaping survival outcomes for patients with large cerebellar metastases. A higher total radiation dose was independently associated with increased mortality (HR 1.17, *p* = 0.043). This suggests that dose escalation in this specific population may reflect more aggressive tumor biology, increased treatment-related toxicity, or suboptimal dose volume relationships within the constrained anatomy of the posterior fossa. The association between higher doses and worse OS may reflect confounding by indication and collinearity between dose and fractionation, as clinicians tailored treatments to baseline risks.

Conversely, a greater number of treatment fractions was significantly associated with improved survival (HR 0.53, *p* = 0.038). This supports the hypothesis that hypofractionated regimens (FSRS) may offer a therapeutic advantage in this setting, where the proximity to critical structures such as the brainstem and the fourth ventricle may limit the safety of high-dose single fraction treatments. These findings align with prior studies demonstrating that radioresistant histologies and high dose single fraction SRS are often associated with inferior survival and higher toxicity [19–21]. At the same time, our results reinforce the growing body of evidence suggesting that hypofractionated approaches can improve the safety profile while maintaining comparable local control, especially for larger lesions [21, 23]. This underscores the rationale for the selective use of FSRS to mitigate toxicity without compromising oncologic efficacy.

### Clinical implications

Overall, the evidence presented in this study supports LINAC-based SRS as an effective, non-invasive treatment modality for large posterior fossa metastases [19–22]. Our findings corroborate prior reports and demonstrate that SRS achieves not only excellent local control and favorable survival outcomes but also meaningful neurological improvement through the reduction of tumor mass and associated peritumoral edema. These benefits are particularly relevant given the substantial morbidity and inherent surgical risks associated with posterior fossa operations [13–15].

Furthermore, the observed significant reduction in tumor volume and the relief of mass effect highlight SRS as a potential alternative to surgical decompression. By inducing significant tumor shrinkage, SRS serves as both an effective disease controlling strategy and a valuable cytoreductive and palliative tool, capable of providing rapid neurological stabilization or improvement.

### Study limitations

Limitations include the retrospective design, potential selection bias, the absence of Biologically Effective Dose (BED) calculations, and lack of a contemporary surgical control group. The small cohort, heterogeneous histologies, and inter-institutional variability in treatment planning further limit definitive conclusions. Additionally, while systemic therapies and radiation techniques evolved over the 17-year study period, this heterogeneity did not significantly impact survival outcomes in our internal era analysis. Incomplete follow-up data for functional status and imaging also constrained secondary analyses. Nonetheless, the two-institution design, well defined volumetric inclusion criteria, and detailed clinical radiological correlations strengthen the generalizability of our findings.

## Conclusion

In this two-institution retrospective cohort, upfront stereotactic radiosurgery for large posterior fossa metastases achieved high rates of local control (93%), significant tumor mass reduction, and favorable survival outcomes, with a median survival exceeding one year. These outcomes were achieved with a remarkably low incidence of severe post-treatment complications requiring surgical resection or shunting (5%), highlighting a favorable safety profile even in this high-risk population.

Our findings suggest that in carefully selected patients, SRS represents a robust primary treatment strategy that can obviate the need for routine surgical resection, even in the setting of large volume posterior fossa disease.

Prospective, comparative trials with surgery are warranted to further refine optimal patient selection and treatment paradigms.

## Supplementary Information

Below is the link to the electronic supplementary material.

Supplementary Material 1

## Data Availability

The datasets generated during and/or analyzed during the current study are available from the corresponding author on reasonable request.
