# Supplementary material for: Upfront stereotactic radiosurgery for large posterior fossa metastases: a multicenter evaluation of clinical outcomes
Source: J Neurooncol. 2026 Mar 2;177(1):25. doi: 10.1007/s11060-026-05483-w (PMC12953285; doi:10.1007/s11060-026-05483-w)

Supplementary Information

Journal Name: Journal of Neuro-Oncology.

Article Title: Upfront Stereotactic Radiosurgery for Large Posterior Fossa Metastases: An Effective Alternative to Surgery

Authors: Ariel Ben-Shoshan, Sami Heymann, José Asprilla, Paz Kelmer, Samuel Moscovici, Yair Hillman, Noam Weitzman, Rotem Bohbot, Anton Wohl, Zvi R. Cohen, Yaacov R. Lawrence, Marc Wygoda, Yigal Shoshan, Tehila Kaisman-Elbaz, and Tal Falick Michaeli

Corresponding Author:

Tal Falick Michaeli, MD, PhD, MBA.

Hebrew University-Hadassah Medical Center

Sharett Institute of Oncology

POB 12272 Jerusalem, Israel 9112002

Email: tal.michaeli@mail.huji.ac.il

ORCID: 0000-0001-7915-4250

Online Resource 1 Representative SRS and FSRS treatment plans showing dose distribution and conformality.

Description: Left: A 5-arc SRS plan delivering 20 Gy in 1 fraction to the 80% isodose line. The plan provides optimal conformality (CI = 1.2) and minimal dose to the organs at risk (OARs). **Right:** A 5-arc FSRS plan delivering 27 Gy in 3 fractions to the 80% isodose line. The plan provides optimal conformality (CI = 1.18) and minimal dose to the OARs.

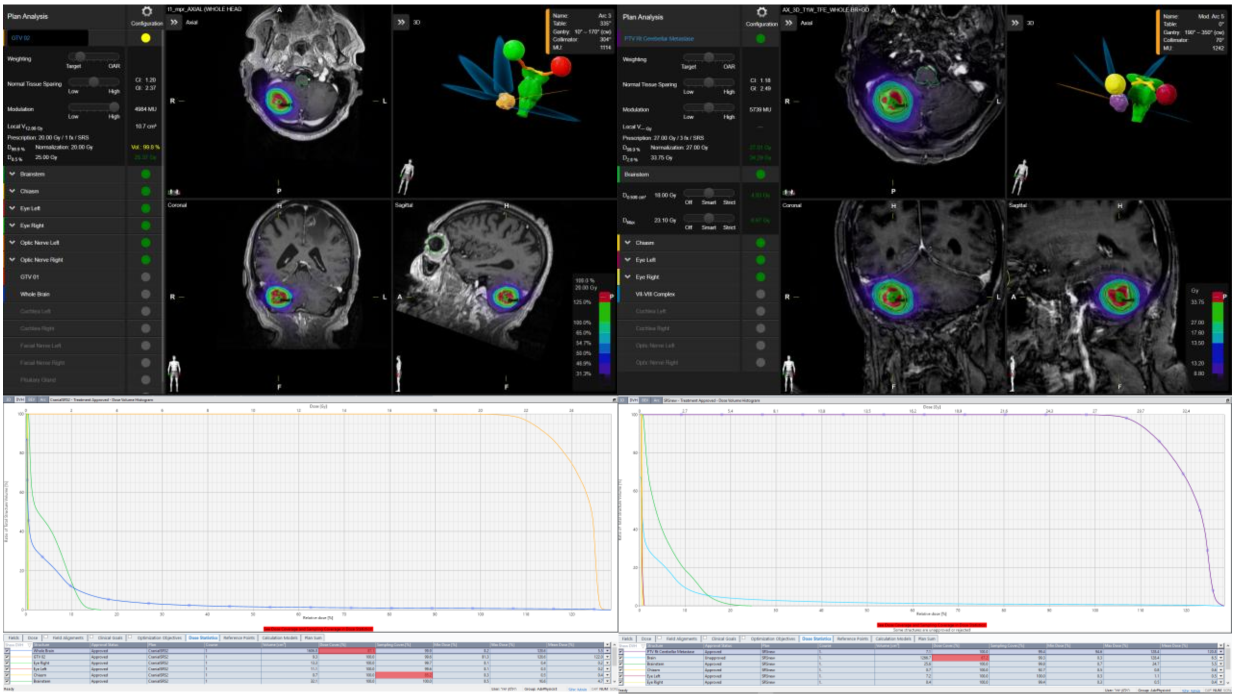

Supplement: Supplementary file 1 — Supplementary Material 1 [file 11060_2026_5483_MOESM1_ESM.pdf]
